# Abnormal Regional Homogeneity and Functional Connectivity of Baseline Brain Activity in Hepatitis B Virus-Related Cirrhosis With and Without Minimal Hepatic Encephalopathy

**DOI:** 10.3389/fnhum.2018.00245

**Published:** 2018-06-22

**Authors:** Qing Sun, Wenliang Fan, Jin Ye, Ping Han

**Affiliations:** ^1^Department of Radiology, Union Hospital, Tongji Medical College, Huazhong University of Science and Technology, Wuhan, China; ^2^Department of Gastroenterology, Union Hospital, Tongji Medical College, Huazhong University of Science and Technology, Wuhan, China

**Keywords:** minimal hepatic encephalopathy, regional homogeneity, functional connectivity, hepatitis B virus-related liver cirrhosis, resting state fMRI

## Abstract

**Background and Aims**: Abnormalities in neural activity have been reported in cirrhosis with minimal hepatic encephalopathy (MHE). However, little is known about the neurophysiological mechanisms in this disorder. We aimed to investigate the altered patterns of regional synchronization and functional connections in hepatitis B virus-related cirrhosis (HBV-RC) patients with and without MHE using both regional homogeneity (ReHo) and region of interest (ROI)-based functional connectivity (FC) computational methods.

**Methods**: Data of magnetic resonance imaging scans were collected from 30 HBV-RC patients with MHE, 32 HBV-RC patients without MHE (NMHE) and 64 well-matched controls. Several regions showing differences in ReHo after one-way analysis of variance (ANOVA) were defined as ROIs for FC analysis. Next, *post hoc t*-tests were applied to calculate the group differences in ReHo and FC (false discovery rate (FDR) correction, *p* < 0.05). Correlations between clinical variables and the altered ReHo and FC were then assessed in patient groups.

**Results**: Across three groups, significant ReHo differences were found in nine ROI regions mainly within the visual network (VN), dorsal attention network (DAN), somatomotor network (SMN), fronto parietal control (FPC) network and thalamus. Compared with healthy controls (HC), the MHE group exhibited abnormal FC mainly between the right calcarine (CAL.R) and middle frontal gyrus (MFG.L)/right thalamus. The MHE patients showed increased FC between the MFG.L and CAL.R compared to NMHE patients. Disease duration of MHE patients was positively correlated with increased mean ReHo values in the right fusiform gyrus (FFG); psychometric hepatic encephalopathy score (PHES) test scores were negatively correlated with increased FC between MFG.L and CAL.R and positively correlated with reduced FC between the CAL.R and THA.R. For NMHE patients, the mean ReHo values in the right frontal pole were positively correlated with disease duration and positively correlated with the PHES scores.

**Conclusion**: Our results exhibited that the functional brain modifications in patients with and without MHE are characterized by compound alterations in local coherence and functional connections in the VN, SMN, DAN, FPC networks and thalamus by using a combination of ReHo and ROI-based FC analysis. These functional imaging changes are correlated with disease duration/PHES. This study helped us gain a better understanding of the features of brain network modifications in cirrhosis.

## Introduction

Minimal hepatic encephalopathy (MHE) is a neurocognitive complication associated with cirrhotic patients that exhibits subtle neuropsychological dysfunction (Zhang et al., 2013), such as mild attention disorder, even after the apparent resolution of over-hepatic encephalopathy (overt HE; Umapathy et al., 2014). Notably, patients with MHE have no obvious clinical manifestations; their neuropsychological impairments are difficult to check out by routine clinical examinations, resulting in a relatively high rate of misdiagnosis (Chen Q.-F. et al., 2016). The psychometric hepatic encephalopathy score (PHES), a battery of psychometric tests, were recommended for diagnosing MHE and to assess MHE-related neuropsychological functions (Duarte-Rojo et al., 2011; Lv et al., 2013a; Wang J.-Y. et al., 2013). However, the results of psychometric tests were often influenced by several confounding factors confounding factors, such as educational level (Weissenborn et al., 2001). Furthermore, MHE is highly prevalent in patients with cirrhosis in China (30%–84%), where the hepatitis B virus remains the primary etiology for liver cirrhosis and approximately 50% of patients with MHE could develop overt HE (Hartmann et al., 2000; Urios et al., 2017). Patients with MHE have poor prognoses causing negative effects on the quality of life of patients, such as an increased risk of vehicular accidents (Tao et al., 2013; Qi et al., 2014). Thus, it is critical to find new biomarkers for MHE diagnosis promoting early treatment and preventing further episodes.

Resting-state functional magnetic resonance imaging (rs-fMRI), a powerful tool for exploring intrinsic functional synchronization (Lee et al., 2013), has been widely used to investigate alterations in brain functional topology in studies of various diseases (Di Martino et al., 2008). The regional homogeneity (ReHo) analysis of rs-fMRI data is an unbiased method to investigate local synchronization in the brain and has been applied to different disorders (Jiang et al., 2015; Chen Q.-F. et al., 2016). The ReHo algorithm was based on the hypothesis that the activity of each voxel within one defined brain region has some similar temporal features to its neighboring voxels (Qi et al., 2012). The regions of interest (ROI)-based functional connectivity (FC) analysis has been extensively used to investigate functional interactions among anatomically separated brain regions, by measuring time series correlation between each pair of the predefined ROI areas in the brain (Wei et al., 2014). Several rs-fMRI studies have reported that patients with MHE have impaired functional interactions in certain networks (Chen et al., 2014; Chen H.-J. et al., 2016). However, one limitation of these studies was that these results were based on a heterogeneous patient cohort with different etiologies of cirrhosis, while etiology could be one confounding factor affecting cerebral function alterations. In addition, the ReHo measure is usually used to characterize spontaneous brain activity at a limited anatomical distance, while the FC is applied to characterize the correlation of the functional patterns of blood oxygen level-dependent signals in between spatially distant brain areas at a network level. Thus, the two analyses, ReHo and FC, are considered to be mutually complementary for detecting both local and remote brain activity synchronization (Cui et al., 2016), and have conjunctively applied in other diseases (Liu et al., 2016). However, for functional networks in hepatitis B virus-related cirrhosis (HBV-RC) patients with or without MHE, abnormalities of regional and inter-regional function interaction patterns have not been well characterized either. To our knowledge, no rs-fMRI studies have simultaneously investigated alterations in the local synchronicity and functional connections of neural activations in a homogeneous cohort of HBV-RC patients with and without MHE, which may give more insights to MHE-related mechanisms than either method alone.

This study aimed to explore the local coherence of intrinsic brain activity and inter-regional connectivity features in patients with and without MHE by using ReHo and ROI-based FC analysis. Based on previous findings that there are abnormalities in both local brain activity and FC in one disease simultaneously (Liu et al., 2016; Lv et al., 2017), we hypothesized that: (1) regional disturbances of ReHo at both cortical and subcortical levels could be detected in HBV-RC patients with or without MHE; (2) the MHE/NMHE patients showed alterations in intrinsic functional connections between different ROI regions pairs; and (3) changes of ReHo and FC in specific functional networks might be correlated with the disease duration or neuropsychological performances measured by the PHES tests.

## Materials and Methods

### Participants

This study was approved by the Medical Ethics Committee at Tongji Medical College of Huazhong University of Science and Technology, and all participants gave written informed consent in agreement with the Declaration of Helsinki. From December 2015 to January 2017, 30 HBV-RC patients with MHE (24 males; mean age: 48.8 ± 12.2 years), 32 HBV-RC patients without MHE (NMHE, 28 males; mean age: 46.3 ± 9.2 years) and 64 healthy controls who were age-, gender- and education-matched (46 males; mean age: 46.8 ± 9.7 years) were recruited; all participants underwent clinical examinations and neuropsychological tests including the PHES tests and Mini-Mental State Examination (MMSE) before the MR scanning. The HBV-RC diagnosis was based on the medical histories, established clinical examinations and biochemical results as well as imaging findings (such as abdominal ultrasonography and MRI) with standard clinical practice guidelines (Ferenci et al., 2002). According to the Child–Pugh classification of liver function, 8, 8 and 14 of the 30 MHE patients as well as 11, 10 and 11 of the 32 NMHE patients had Child–Pugh grade A, B and C, respectively.

The inclusion criteria for recruitment of all patients were as follows: patients with Hepatitis B cirrhosis; the completion of the MMSE, PHES tests and the MRI scanning without any MRI contraindication; right-handedness. There were no control subjects with any types of liver disease. Exclusion criteria for all subjects included other types of virus-related cirrhosis, any carcinoma, severe metabolic diseases (e.g., thyroid dysfunction), drug intoxications, psychiatric or neurological diseases, head injury, positive human immunodeficiency virus status, bad vision, current overt HE or history of clinical manifestation of overt HE, taking psychotropic medications, probable dementia (MMSE scores ≤24; Cui et al., 2016), left-handedness, any MRI contraindication, and a translation of more than 2.0 mm or a rotation of more than 2.0° during the MRI scan (Chen et al., 2015).

### Neuropsychological Test

The PHES, recommended by a commission of experts on the hepatic encephalopathy as test batteries suitable for the diagnosis of MHE (Randolph et al., 2009), is a normative set of five psychometric tests for assessing neurocognitive functions (such as motor, visual perception, memory and attention) in cirrhotic patients (Duarte-Rojo et al., 2011; Seo et al., 2012; Li et al., 2013). Five neuropsychological tests were completed by all participants: (1) the digit-symbol test (DST); (2) serial dotting test (SDT); (3) number connection test A (NCT-A); (4) number connection test B (NCT-B); and (5) line tracing test (LTT); PHES has been validated for identifying patients with MHE as reported in many previous studies in different countries (Duarte-Rojo et al., 2011; Seo et al., 2012). The SDT and LTT were available on the network of Hepatic Encephalopathy^1^. The DST was a subtest of the Wechsler Adult Intelligence Scale-Revised for China (WAIS-RC). Because several participants did not understand the English alphabet, the English characters from A to L in the NCT-B were replaced with Chinese characters from 1 to 12 in the same order (Li et al., 2013). The NCT-A, the China Revised NCT-B, and the DST tests have also been validated and widely applied for diagnosing MHE in patients with cirrhosis in China (Zhong et al., 2001; Chen et al., 2012b; Lv et al., 2013a). A trained and experienced doctor performed the PHES tests on all enrolled subjects. The healthy controls were included in the five tests to gain a PHES “normative or reference value.” Regression models and formulas were obtained with Pearson’s correlations between the psychological tests scores and age as well as education level by applying multivariate linear regression analyses (Duarte-Rojo et al., 2011; Lv et al., 2013a). These regression formulas were then applied to predict values for patients. The difference between the observed and predicted test scores was divided by SDs for the reference group. Differences for each test were summarized in the following: the observed result ≥1 SD predicted values was scored as +1 points, results −1 SD and −2 SDs < the predicted were scored as −1 and −2 respectively as well as the observed ≤−3 SDs was scored as −3 points (Duarte-Rojo et al., 2011; Lv et al., 2013a). In all cirrhotic patients with no evidence of overt HE and no previous history of overt HE, those having a PHES score of ≤−4 points were considered to have MHE and others having a PHES score of >−4 points were considered to be without MHE (Duarte-Rojo et al., 2011; Lv et al., 2013a).

### MRI Data Acquisition

All MRI data were acquired on a 3.0T GE Discovery MRI 750 w (Grandview Blvd, Waukesha, WI, USA) using an eight-channel head coil. Tight foam padding was fixed to keep their head still and earphones were used to reduce scanner noise during the scan. Each subject was instructed to relax, lie quietly, keep their eyes closed but not fall asleep, not think of anything in particular, and remain still during the MRI scan. The rs-fMRI images were obtained by using an echo-planar imaging sequence. The rs-fMRI acquisition parameters were as follows: repetition time (TR), 3000 ms; echo time (TE), 30 ms; flip angle, 90°; matrix, 64 × 64; field of view (FOV), 256 mm × 256 mm; section thickness, 4.0 mm; number of sections, 40; and NEX, 1.0. The total scan time was 10 min. Axial T2-weighted fluid-attenuated inversion recovery (FLAIR) images(inversion time (TI), 2200 ms; TE/TR, 120/12,000 ms; and matrix, 320 × 224) and three-dimensional brain volume imaging (3D BRAVO) images (TI, 400 ms; TR, 9.1 ms; TE, 3.5 ms; FOV, 25.6 × 25.6 cm^2^; matrix, 256 × 256; 180 slices; slice thickness, 1.1 mm) were applied at the same orientation for acquiring the anatomical images. All of the images were evaluated by two experienced radiologists blinded to the group status.

### Data Preprocessing

Functional images preprocessing was performed by using the Data Processing and Analysis of Brain Imaging (DPABI) toolbox (Yan et al., 2016). The first 10 time points were discarded for adaptation of all participants to the scanning environment and scanner calibration. The remaining volumes were used for further data preprocessing, which included slice-timing correction and realignment correction for head movements. Any subject who had a maximum displacement in any of the three cardinal directions (x, y, z) >2.0 mm or a maximum spin (x, y, z) >2.0° was excluded from the study (Chen et al., 2015). Next, the resulting functional data were spatially normalized to the Montreal neurological institute (MNI) space and resampled to 3 × 3 × 3 mm^3^. Then, linear-trend discarding and temporal band-pass filtering (0.01 Hz < *f* < 0.08 Hz) were performed to reduce the effects of low- frequency drifts and high-frequency physiological noise. Several nuisance variables including head motion parameters, white matter and cerebrospinal fluid signals were regressed out were regressed out. We took the Friston 24-parameter model to remove artifacts caused by head motion (Yan et al., 2013). Regarding the global signal, previous studies have demonstrated that removing it may lead to enhances in negative correlations (Murphy et al., 2009; Weissenbacher et al., 2009) or affect differences in functional connection (Saad et al., 2013). Given its controversial biological interpretations, the global signal was not regressed out, which has been applied in prior studies (Murphy et al., 2009; Wang J. et al., 2013; Luo et al., 2015).

### Computation of ReHo Maps

Following previous calculation procedures (Ni et al., 2012; Yan et al., 2016), we computed ReHo maps of all subjects by computing the Kendall coefficient of concordance (KCC; Zang et al., 2004). The KCC was used to measure the ReHo of the time series within one voxel and its most adjacent 26 neighboring voxels in a voxel-wise manner. A whole brain map of ReHo values for each subject was calculated. We normalized ReHo values by dividing the ReHo value of each voxel in one ReHo map by the mean whole-brain ReHo value, and then obtained the mean ReHo (mReHo) maps for each subject (Zhang et al., 2012). Then, all mReHo maps were smoothed with a Gaussian kernel of 4-mm full width at half maximum (FWHM; Cui et al., 2016). A one-way analysis of variance (ANOVA) was used on the mReHo maps to identify regions with significant differences among the three groups (*p* < 0.05, false discovery rate (FDR) correction with a minimum cluster size of at least 10 contiguous voxels; Mutschler et al., 2010; Plaza et al., 2012). These areas were then extracted as a mask. The *post hoc* *t-test* of the ReHo maps within this mask was used between each pair of the three groups (MHE vs. NMHE, MHE vs. healthy controls (HC), NMHE vs. HC), with nuisance covariates, i.e., age, education, gender and head motion. The threshold of the tests was set at *p* < 0.05 with FDR correction for multiple comparisons using the DPABI software.

### ROI-Based Functional Connectivity Analysis

Based on the ReHo results after the one-way ANOVA test among the three groups, we chose nine brain regions with significant group differences obtained in ReHo as the ROIs for the ROI-based FC analysis: the right fusiform gyrus (FFG.R), right superior frontal gyrus, orbital part (SFGorb.R), right thalamus (THA.R), right calcarine (CAL.R), left middle frontal gyrus (MFG.L), left precentral gyrus (PreCG.L), right postcentral gyrus (PoCG.R), right superior frontal gyrus (SFG.R) and left paracentral lobule (PCL.L; FDR-corrected, *p* < 0.05). To uncover the functional connections patterns among these regions, we constructed their pair-wise connectivity matrix individually using the FC analysis; FC analysis calculates the functional correlations between any of each of the two ROI mean time courses among different ROI regions using the DPABI software. For each ROI region of nine ROIs, mean time series of all ROIs were calculated for each subject and then correlated with the other eight ROIs. We obtained 9 × 8/2 ROI correlation coefficients for each subject; the Fisher’s Z transformation was applied in all correlation coefficient matrixes to enhance the normality for comparisons (Tang et al., 2011). Next, for comparing the FC, we performed the one-way ANOVA test and *post hoc t*-tests to calculate the group differences of *z* values of ROI correlation coefficients across the three groups. The threshold of all tests was set at *p* < 0.05 with FDR correction for multiple comparisons using the GRETNA software toolbox (Wang et al., 2015). Alternatively, a threshold of *p* < 0.001 (uncorrected for multiple comparisons) was applied in the condition group comparisons based on the stringency of the group contrasts used in this exploratory study.

### Statistics Analysis

The Kolmogorov-Smirnov test, one-way ANOVA test and *post hoc t*-tests, two independent samples *T*-test, χ2 test and the Mann-Whitney U test were performed to analyze the group differences in the demographic and clinical data using SPSS 18.0 software (SPSS Inc., Chicago, IL, USA). Multivariate linear regression was applied to calculate the PHES normative data (Seo et al., 2012). The significance level was set at *p* < 0.05.

### ReHo, FC and Correlation Analyses

The clusters showing significant group differences in ReHo and FC between each pair of the three groups (MHE vs. NMHE, MHE vs. HC, NMHE vs. HC) were visualized with the BrainNet Viewer (Xia et al., 2013) and DPABI software viewer (FDR-corrected, *p* < 0.05). The mean ReHo values and FC correlation coefficient values of these abnormal clusters were extracted using DPABI software. Then, we applied Pearson’s correlation analysis to examine the relationship between altered ReHo/FC and with clinical variables (e.g., PHES tests scores and disease duration) in the MHE/NMHE patients. Considered this is an exploratory study, correlation analyses were not corrected by Bonferroni correction.

## Results

### Demographics and Clinical Data

The demographic and clinical characteristics for all patients and healthy subjects are shown in Table 1. There were no significant differences in age, gender or education among the three groups. All participants in our study performed normally on the MMSE test.

**Table 1:** Demographic and clinical characteristics in cirrhotic patients with or without minimal hepatic encephalopathy (MHE) and normal controls.

Demographic	MHE (*n* = 30)	NMHE (*n* = 32)	Control (*n* = 64)	*p*-value
Gender (male/female)	24/6	28/4	46/18	*p* = 0.210^a^
Age (years)	48.8 ± 12.2	46.3 ± 9.2	46.8 ± 9.7	*p* = 0.577^b^
Education level (years)	7.0 ± 4.2	7.6 ± 4.8	9.2 ± 3.8	*p* = 0.054^b^
Disease duration (years)	9.8 ± 5.8	6.8 ± 4.9	−	*p* = 0.029^c,^*
Child–Pugh’s class: A/B/C (n)	8/8/14	11/10/11	−	−
MMSE (score)	27.8 ± 1.1	28.2 ± 1.1	29.1 ± 1.1	*p* < 0.001^b,^*
PHES (scores)	−8.0 ± 3.1	0.2 ± 1.0	−0.2 ± 1.4	*p* < 0.001^b,^*

All values are displayed as the mean ± SD. *Significant differences (p < 0.05). ^a^The P value for gender distribution was obtained by chi-square test. ^b^The P value for age, education level and neuropsychological tests scores was obtained by one-way analysis of variance. ^c^The P value for disease duration was obtained by independent t-tests. Abbreviations: MHE, minimal hepatic encephalopathy; NMHE, without minimal hepatic encephalopathy; MMSE, Mini-Mental State Examination; PHES, psychometric hepatic encephalopathy score.

### Psychometric Test Results

Compared with the PHES in healthy controls (mean = −0.19; SD = 1.42; range: −2 to +3), HBV-RC patients with MHE had significantly worse PHES scores (mean = −8.00; SD = 3.10; range: −13 to −4). The 95% range of the normative values between the mean −2 SDs and mean +2 SDs was −3.03 to 2.65 points. The MHE was diagnosed when the PHES score was ≤−4 points (Li et al., 2013).

### Regional Homogeneity (ReHo) Differences Between Groups

**Figure 1:**
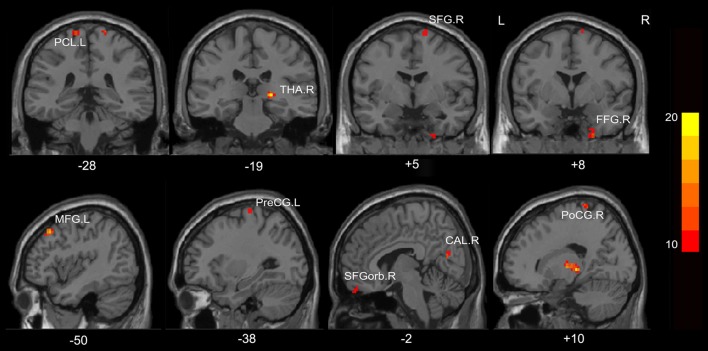
Regions showing significantly different regional homogeneity (ReHo) across the three groups (*p* < 0.05, false discovery rate (FDR) corrected). FFG, fusiform gyrus; SFGorb, superior frontal gyrus, orbital part; PoCG, postcentral gyrus; PreCG, precentral gyrus; THA, thalamus; CAL, calcarine; SFG, superior frontal gyrus; PCL, paracentral lobule; MFG, middle frontal gyrus; R, Right; L, Left. The color bar indicates a scale of *T* values.

A one-way ANOVA test showed significant group differences in ReHo among the MHE, NMHE and HC groups in the following nine cortical and subcortical regions: FFG.R, SFGorb.R, MFG.L, THA.R, CAL.R, PoCG.R, PreCG.L, SFG.R and PCL.L (FDR-corrected *p* < 0.05; Figure 1, Table 2).

**Table 2:** A one-way analysis of variance result of the regional homogeneity differences among healthy controls and patients with and without MHE.

Brain regions	Peak MNI, mm			*T* score
	*X*	*Y*	*Z*	
Right fusiform gyrus	24	0	−45	11.4
Right thalamus	21	−27	0	16.1
Right calcarine	6	−69	18	12.8
Right superior frontal gyrus, orbital part	9	54	−27	11.0
Left precentral gyrus	−30	−24	72	14.5
Right postcentral gyrus	18	−36	78	11.7
Left middle frontal gyrus	−42	27	45	17.4
Left paracentral lobule	−15	−36	75	11.6
Right superior frontal gyrus	15	−3	75	14.0

*The threshold was set p < 0.05 (false discovery rate correction). MNI, Montreal Neurological Institute*.

**Figure 2:**
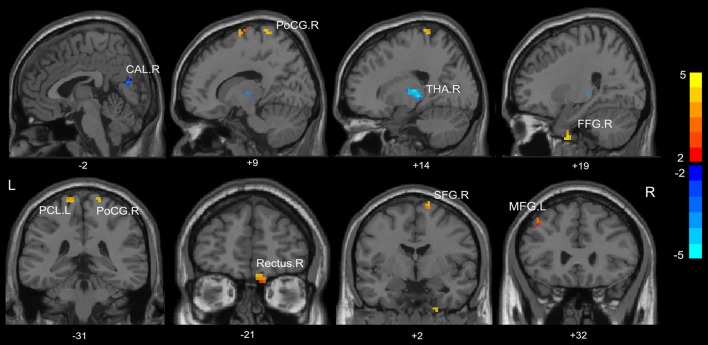
Group differences of mean ReHo between patients with minimal hepatic encephalopathy (MHE) and healthy controls (*p* < 0.05, FDR corrected). FFG, fusiform gyrus; PoCG, postcentral gyrus; THA, thalamus; CAL, calcarine; THA, thalamus; SFG, superior frontal gyrus; PCL, paracentral lobule; MFG, middle frontal gyrus; R, Right; L, Left. The color bar indicates a scale of *T* values.

**Figure 3:**
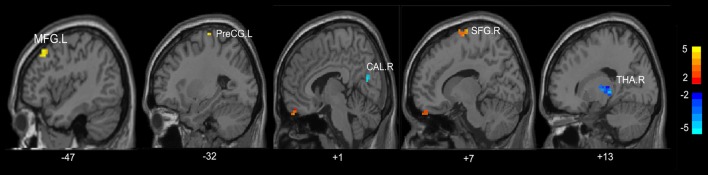
Group differences of mean ReHo between patients without MHE and healthy controls (*p* < 0.05, FDR corrected). PreCG, precentral gyrus; THA, thalamus; CAL, calcarine; SFG, superior frontal gyrus; MFG, middle frontal gyrus; R, Right; L, Left. The color bar indicates a scale of *T* values.

Compared with HC, the MHE patients had significantly altered ReHo mainly in the visual network (VN, FFG.R, CAL.R), somatomotor network (SMN, PoCG.R, PCL.L), frontal parietal control (FPC) network (SFG.R) and THA.R (FDR-corrected *p* < 0.05; Figure 2, Table 3). For NMHE vs. HC, we found a significant ReHo decrease in the THA.R and CAL.R, as well as ReHo enhancement in the right frontal pole, dorsal attention network (DAN, MFG.L), SMN (PreCG.L), and SFG.R in NMHE patients (FDR-corrected *p* < 0.05; Figure 3, Table 3). MHE patients showed increased mean ReHo in the PoCG.R compared to the NMHE group (*p* < 0.001, uncorrected; Table 3).

**Table 3:** Brain areas showing different regional homogeneity between each pair of the three groups.

Brain regions	Peak MNI, mm			*T* score
	*X*	*Y*	*Z*
**MHE vs. Controls (FDR corrected, *p* < 0.05)**				
Right fusiform gyrus	24	0	−49	5.11
Right rectus	6	51	−21	4.25
Right thalamus	21	−26	0	−5.83
Right superior frontal gyrus	15	−3	75	4.43
Left paracentral lobule	−15	−36	75	4.49
Right postcentral gyrus	12	−45	78	4.59
Right calcarine	3	−69	15	−3.77
**NMHE vs. Controls (FDR corrected, *p* < 0.05)**				
Right frontal pole	6	54	−27	4.22
Right superior frontal gyrus	12	−12	78	4.51
Right thalamus	18	−27	0	−4.15
Right calcarine	6	−69	18	−4.84
Left precentral gyrus	−27	−21	72	5.18
Left middle frontal gyrus	−42	30	45	5.60
**MHE vs. NMHE (uncorrected, *p* < 0.001)**				
Right postcentral gyrus	18	−39	75	3.53

*The threshold was set p < 0.05. FDR, false discovery rate; MNI, Montreal Neurological Institute; MHE, minimal hepatic encephalopathy; NMHE, without minimal hepatic encephalopathy*.

### Functional Connectivity (FC) Groups Differences

**Figure 4:**
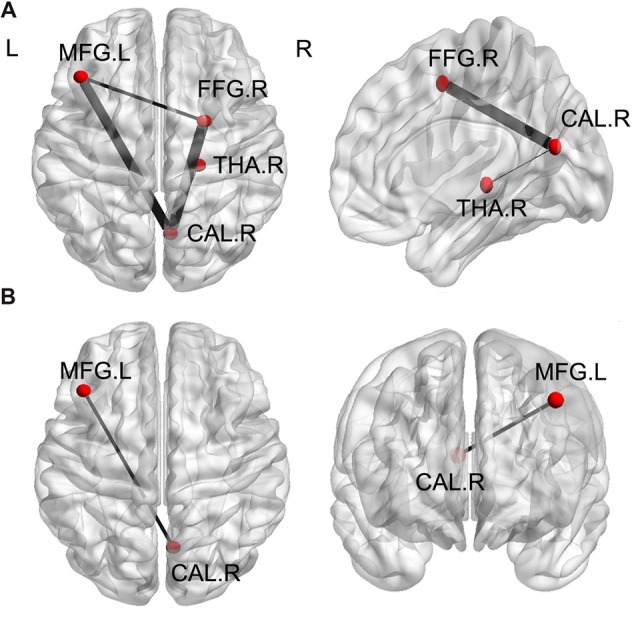
Significant differences of functional connectivity (FC) of ROI regions among the three groups (*p* < 0.05, FDR corrected).** (A)** Compared with healthy controls, the MHE patients showed significantly lower FC between the CAL.R and FFG.R/THA.R, as well as greater FC between the MFG.L and FFG.R/CAL.R. **(B)** When compared to the NMHE group, greater FC between CAL.R and MFG.L in MHE patients. The threshold was set *p* < 0.05 with FDR correction. Lines represent FC between each pair of defined ROI regions with significant group differences between two groups. The size of lines represents the absolute value of *T* values. FFG, fusiform gyrus; THA, thalamus; CAL, calcarine; MFG, middle frontal gyrus; R, Right; L, Left.

A one-way ANOVA test exhibited significant group differences in FC among the three groups (FDR-corrected *p* < 0.05). As is shown in Figure 4A and Table 4, compared with HC, the MHE patients showed significantly lower FC between the CAL.R and FFG.R/THA.R, as well as greater FC between the MFG.L and FFG.R/CAL.R (FDR-corrected *p* < 0.05). We detected significantly greater FC between CAL.R and MFG.L in MHE patients compared to the NMHE group (FDR-corrected *p* < 0.05; Figure 4B; Table 4). When compared to HC, significantly altered FC in NMHE patients were not observed in the present study after FDR correction.

**Table 4:** Brain regions with significant group differences on functional connectivity (FC) between patients with and without MHE and healthy controls (HCs).

ROI region	Connected brain region	*T*-value
**MHE vs. Controls**		
**(FDR corrected, *p* < 0.05)**		
Right calcarine	Left middle frontal gyrus	2.606
	Right thalamus	−2.007
	Right fusiform gyrus	−2.673
Left middle frontal gyrus	Right fusiform gyrus	2.218
**MHE vs. NMHE**		
**(FDR corrected, *p* < 0.05)**		
Right calcarine	Left middle frontal gyrus	2.240

*ROI, region of interest; FDR, false discovery rate; MHE, minimal hepatic encephalopathy; NMHE, without minimal hepatic encephalopathy*.

### Correlations of the Abnormal Reho and FC With Clinical Variables in MHE

**Figure 5:**
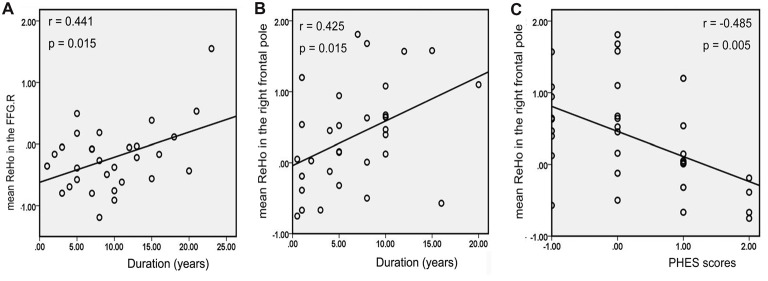
Correlation between abnormal regional homogeneity (ReHo) and clinical variables in cirrhotic patients with and without MHE. Panel **(A)** depicts mean ReHo in the right fusiform gyrus (FFG.R), which was positively correlated with the disease duration of patients with MHE; panel **(B)** shows mean ReHo in the right frontal pole, which was positively correlated with the disease duration in the patients without MHE (NMHE); panel **(C)** depicts negative correlations between mean ReHo in the right frontal pole and psychometric hepatic encephalopathy scores (PHES) in the NMHE group. The threshold was set *p* < 0.05 without multiple comparison correction.

**Figure 6:**
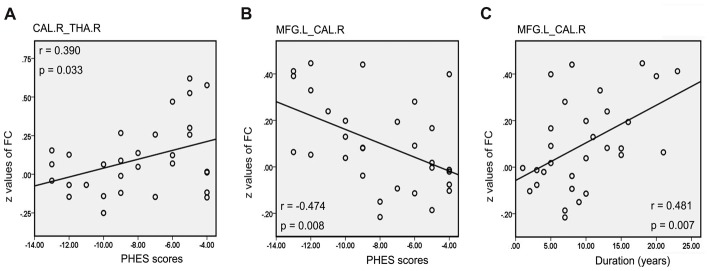
Correlation between abnormal FC and clinical variables in cirrhotic patients with MHE. Significantly reduced *z* values of FC between the right calcarine (CAL.R) and right thalamus (THA.R) in the MHE group were positively correlated with poor PHES performance **(A)**. Within the visual network (VN) and dorsal attention network (DAN), the increased *z* values of FC between the CAL.R and MFG.L in MHE patients were negatively correlated with the PHES test scores **(B)**, but also negatively correlated with the disease duration **(C)**. A threshold of *p* < 0.05 was set without multiple comparison correction.

There was a positive correlation between the increased mean ReHo values in FFG.R and disease duration in patients with MHE (*r* = 0.441, *p* = 0.015; Figure 5A). For NMHE patients, the mean ReHo values in the right frontal pole were not only positively correlated with disease duration (*r* = 0.425, *p* = 0.015; Figure 5B) but also negatively correlated with the PHES scores (*r* = −0.485, *p* = 0.005; Figure 5C). Significantly reduced *z* values of FC between the CAL.R and THA.R in MHE were also positively correlated with poor PHES performance (*r* = 0.390, *p* = 0.033; Figure 6A). Within the VN and DAN, the increased *z* values of FC between the CAL.R and MFG.L in MHE patients were not only negatively correlated with the PHES test scores, in which a lower score suggested worse neuropsychological performance (*r* = 0.474, *p* = 0.008; Figure 6B) but also negatively correlated with the disease duration (*r* = 0.481, *p* = 0.007; Figure 6C). The abovementioned correlations were not corrected for multiple comparisons, due to the relatively small-size patient group and the exploratory feature of this correlation analysis.

## Discussion

This study is the first attempt to simultaneously investigate the altered patterns of both local coherence and functional connections altered patterns in HBV-RC patients with and without MHE by combining both ReHo and ROI-based FC analyses. We demonstrate that HBV-RC patients with or without MHE show abnormalities in subcortical and cortical functional networks, and some of these alterations are associated with clinical characteristics. The present findings provide more information on the underlying pathophysiology mechanism of HBV-RC patients with MHE/NMHE in relation to distributed networks dysfunctions.

### Regional Homogeneity Alterations in MHE/NMHE Patients

Altered resting-state neural activity is considered to relate to functional disturbances in cirrhosis with and without MHE. Several previous rs-fMRI studies have revealed that some brain regions with disturbed local coherence are involved in the disease progress in MHE or NMHE, including cortical regions of the frontal cortex, PoCG and subcortical regions of the thalamus, which were consistent with our results (Qi et al., 2012, 2013; Lv et al., 2013a; Chen Q.-F. et al., 2016). Compared with HC, the cirrhotic patients with or without MHE exhibited increased mean ReHo in the FFG.R and PoCG.R, and decreased mean ReHo in the CAL.R, which indicate aberrant brain activity synchronization in these brain areas. In the VN, the CAL and FFG play essential roles in visional association functions, such as visual attention, processing and discrimination (Lee et al., 2000; Lv et al., 2013b). The PreCG and PoCG (components of the SMN) are primarily involved in motor control. Therefore, the disturbed regional synchronization of the VN and SMN may account for the visual and motor dysfunction in the MHE/NMHE patients, which were also reported in prior studies (Lockwood et al., 2002; Schiff et al., 2005; Weissenborn et al., 2007; Ni et al., 2012; Qi et al., 2012). Some positron emission tomography studies also displayed abnormal cerebral glucose utilization in the visual association regions (Lockwood et al., 2002), and motor-related regions (Weissenborn et al., 2007) in cirrhosis. Moreover, in line with previous neuroimaging studies in cirrhosis (Chen et al., 2012b; Lv et al., 2013a; Ni et al., 2014), MHE/NMHE patients showed enhanced mean ReHo in the DAN (MFG.L) and SFG.R compared with healthy controls. Attention and cognitive control deficits are also regarded as MHE-related characteristics (Bajaj et al., 2009; Chen et al., 2014), and can affect working memory and learning ability (Amodio et al., 2005; Felipo et al., 2012). The DAN is associated with the orienting of attention (Corbetta and Shulman, 2002; Chen et al., 2014), and the SFG within the FPC network is involved in cognitive control (du Boisgueheneuc et al., 2006; Kompus et al., 2009; Gao and Lin, 2012). Therefore, we speculate that altered local synchronization in the DAN and FPC network regions may account for the attention and cognition deficits in MHE/NMHE. In addition, MHE patients exhibited increased mean ReHo in the SMN (PoCG.R; voxel *p* < 0.001, uncorrected) compared with the NMHE group, indicating further motor dysfunction with the disease progression from NMHE to MHE; this finding was similar to one previous study (Ni et al., 2012). The result of group differences in ReHo between the two patient groups is exploratory because they did not pass multiple comparisons correction. Considering that the sample size for patient groups is relatively small and the variations in disease duration, we do not exclude the possibility that these factors might influence the validity of the statistical analysis and findings of the present study.

A striking finding was the identification of a positive correlation between the PHES tests scores and the enhanced mean ReHo values in the VN (FFG.R) in MHE patients, which was in line with a previous study (Chen et al., 2012a). For NMHE patients, the increased mean ReHo values in the prefrontal cortex (right frontal pole) were correlated with both the PHES scores and disease duration. PHES, a set of the neuropsychological tests, was widely used to assess the neurocognitive function in cirrhotic patients (Giménez-Garzó et al., 2017). The prefrontal cortex plays an essential role in cognitive control (Miller and Cohen, 2001). These correlations suggest that the ReHo indicator in these brain regions may be a potential biomarker to reflect neuropsychological alterations in cirrhosis and the ReHo in the right frontal pole may reflect the clinical progression of NMHE.

### ROI-ROI Functional Connectivity Alterations in MHE/NMHE Patients

There are distant and local interconnections between different regions, shaping brain networks. To our knowledge, regional synchronization and functional connection pattern alterations in MHE/NMHE have not been fully studied in prior rs-fMRI studies at the same time. Thus, the current FC results may extend and confirm recent neuroimaging findings in patients with and without MHE.

When compared with healthy controls, MHE patients exhibited functional connections abnormalities mainly between the VN (CAL.R) and some networks, including the VN, DAN and THA.R, which are in line with previous studies that disruption of the DAN, VN and thalamic functional connections have been reported in MHE (Qi et al., 2012, 2013, 2015). These results suggest that aberrant cooperation of their corresponding function, such as attention control and visual cognition, were disturbed in MHE. Furthermore, the MHE group showed increased FC between the DAN (MFG.L) and VN (CAL.R) compared to NMHE patients. This finding indicates that long-range FC between the two networks had progressively increased functional connections from NMHE to MHE, and the FC values in those regions might be a potential marker for assessing the disease progression but be unspecific for the presence of MHE. Additionally, it has been detected cirrhotic patients’ disturbances in attention control, visual processing, memory, and other cognitive functions (Weissenborn et al., 2001; Weissenborn, 2008). The thalamus is an important subcortical gray matter area, and the thalamic FC network plays an essential role in integrating information across the brain circuits (Qi et al., 2013). Therefore, alterations in the FC within the thalamus, VN and DAN regions may be associated with the impaired attention and visual-spatial capabilities in patients with or without MHE. In addition, it is worth noting that there was no significant difference in FC between NMHE patients and the HC group. The possible reason is that functional connections in NMHE patients might be intact and robust due to functional compensatory or reorganization of the neural mechanism in the brain. However, this result was not in accordance with one prior neuroimaging study by Chen et al. (2014) in which NMHE patients showed an abnormality of FC in the attention-related network compare with controls. One reason for the difference of FC results in NMHE may be the inconsistency of admission criteria. The etiologies of cirrhosis in their study include alcoholic and HBV-related cirrhosis. Some studies suggested that the brain was very susceptible to alcohol action, and consequently low exposure to moderate alcohol could affect neural function (Baglietto et al., 2011). Therefore, we believe that alcohol is a potential confounder in the analysis of FC, which might lead to the results of our two studies being different. Furthermore, we found the correlations between clinical variables and aberrant FC in the VN, DAN and thalamus, which were consistent with prior studies (Qi et al., 2012, 2013). The PHES test scores in MHE patients were negatively correlated with the enhanced FC between the DAN (MFG.L) and VN (CAL.R) and positively correlated with FC between the VN (CAL.R) and THA.R; these correlations indicate that aberrant FC within those networks in MHE patients may be associated with dysfunctions in visual processing, attentive activity and information integration during cognitive tasks. For NMHE patients, FC values between the CAL.R and MFG.L were positively correlated with the disease duration, suggesting that functional connection impairments between the VN and DAN may represent the progression of the disease.

### Limitations

The current study has several limitations. First, the group differences in the ReHo were found in the PoCG.R between the MHE and NMHE groups (voxel *p* < 0.001 uncorrected), while they were no longer significant after FDR correction. We speculate that the current study is a cross-sectional study with a relatively small sample size, which may affect the statistical power and the interpretation of the final results. Therefore, further longitudinal studies with larger groups of participants are needed to further confirm our findings and provide additional information about the abnormalities of the functional network altered patterns with the progression of MHE and NMHE. Second, although we instructed all participants to stay still during the rs-fMRI scan, we cannot completely eliminate the possibility of some uncontrolled factors, such as involuntary head movement, affecting our results during the scanning. Third, the results of abnormal ReHo/FC correlation to clinical variables are exploratory because they did not pass stringent Bonferroni correction, and they thus need to be validated in large-size subjects. Fourth, in order to ensure the homogeneity of the case, all of the patients recruited in our study were HBV-RC subjects with MHE/NMHE. Therefore, our findings may not be generalized to other type of cirrhosis, such as the autoimmune cirrhosis.

## Conclusion

The present study exhibited that the functional brain modifications in MHE and NMHE patients are characterized by compound alterations in local coherence and functional connections in the VN, SMN, DAN, FPC networks and thalamus by using ReHo and ROI-based FC analysis. These functional neuroimaging alterations are correlated with the neuropsychological measures, which provide information on the pathophysiological mechanisms underlying neuropsychological alterations in HBV-RC cirrhosis with or without MHE.

## Author Contributions

QS was involved in the acquisition, analysis and interpretation of data, as well as drafting the manuscript. WF assisted with data acquisition and analysis. PH and JY were involved in revising the manuscript. All authors read and approved this manuscript.

## Conflict of Interest Statement

The authors declare that the research was conducted in the absence of any commercial or financial relationships that could be construed as a potential conflict of interest.

## Footnotes

^1^http://www.redeh.org/
